# Endoscopic papillectomy for ampullary adenomas in familial adenomatous polyposis

**DOI:** 10.1007/s10689-025-00502-7

**Published:** 2025-11-04

**Authors:** C. Wehling, Y. Eckstein, A. Stenzinger, N. Ganion, T. Bruckner, P. Sauer, C. C. Zhang-Hagenlocher, M. Kantowski, R. Koschny

**Affiliations:** 1 Interdisciplinary Endoscopy Center (IEZ)/Department of Gastroenterology, University Hospital Heidelberg, Im Neuenheimer Feld 410, 69120 Heidelberg, Germany; 2 Institute of Pathology, University Hospital Heidelberg, Heidelberg, Germany; 3 Department of Anesthesiology, University Hospital Heidelberg, Heidelberg, Germany; 4 Institute for Medical Biometry, University Hospital Heidelberg, Heidelberg, Germany; 5 Department of General, Visceral, and Transplantation Surgery, University of Heidelberg, Heidelberg, Germany; 6 Department of Gastroenterology, Section Translational Endoscopic Research, University Hospital Heidelberg, Heidelberg, Germany

**Keywords:** Familial adenomatous polyposis coli, Ampullary cancer, Endoscopic mucosal resection, Pancreatitis, Papilla of vater, Endoscopic papillectomy

## Abstract

Objectives of the study were to determine the success, complication and recurrence rate of endoscopic papillectomies in familial adenomatous polyposis (FAP) patients. Ampullary adenoma is frequent in familial adenomatous polyposis (FAP) patients. Endoscopic papillectomy is technically demanding for laterally spreading ampullary adenomas and bleeding, perforation and pancreatitis represent typical complications. 40 FAP patients undergoing endoscopic papillectomy were retrospectively analyzed. Data on periprocedural complications, histopathology, resection techniques and success were collected. Endoscopic papillectomy was performed in 21 male and 19 female patients. Mean adenoma size was 14 mm (8–40 mm). Additional mucosal resection was performed in 45%. Immediate and delayed bleeding occurred in 33% (n = 13) and 13% (n = 5), respectively. Bleeding was associated with male sex and pancreatic endoprosthesis. Endoscopically manageable perforation occurred in 2 patients. Acute pancreatitis occurred in 25% (n = 10) and was significantly associated with female sex. Histopathology revealed papillitis in 5% (n = 2), adenoma with low-grade dysplasia in 73% (n = 29), high-grade dysplasia in 13% (n = 5), and adenocarcinoma in 5% (n = 2). Cumulative recurrence-free resection was achieved in 94% (n = 33/35) after a mean of 1.4 procedures at a median follow-up of 591 days (range 15-2605). Endoscopic papillectomy shows a high clinical success in FAP patients even with laterally spreading adenoma. Adenoma recurrence can be successfully treated with a limited number of reinterventions.

## Introduction

Ampullary adenomas are found in up to 15% of FAP patients [1]. Adenoma size > 10 mm, high-grade dysplasia, and villous histology constitute risk factors for progression, although the general rate of malignant transformation of ampullary adenomas seems to be low in FAP patients [2]. Furthermore, high recurrence and adverse event rates of up to 48% and 30%, respectively, have been reported for endoscopic papillectomy [3, 4]. Therefore, European guidelines recommend endoscopic papillectomy in FAP patients for ampullary adenomas equal to or larger than 10 mm or when high-grade dysplasia or villous histology is present [5, 6]**.** Outcome data after endoscopic papillectomy is very heterogenous in FAP patients and might differ from resections of sporadic ampullary adenomas [7]. In FAP patients, variable extent of lateral adenoma spread and adjacent non-ampullary duodenal adenomas necessitate additional endoscopic resection procedures and thereby impact the outcome of papillectomy. However, recent studies demonstrate similar final success and complication rates for endoscopic resection of sporadic and FAP-associated ampullary adenomas, whereas FAP patients had longer procedures with less en-bloc resections [4]. Alternatively, transduodenal surgical ampullectomy in patients with sporadic duodenal adenoma provides a higher rate of complete resection for the cost of an increased complication rate [8]. Data on FAP patients are comparably scarce. Furthermore, there is no consensus on the optimal endoscopic resection strategy in FAP patients with laterally spreading adenomas involving the papilla, as non-ampullary EMR requires submucosal injection whereas en-bloc papillectomy should be performed without [9]. While some authors report on a two-stage approach with secondary papillectomy weeks after EMR of non-ampullary parts [10], most centers favor a resection in one single session with either EMR or papillectomy first [11]. A 50% risk of periprocedural bleeding has been reported in these patients [12]. Current guidelines on endoscopic papillectomy emphasize that data are still limited and coming from referral centers only [9]. We, therefore, analyzed all consecutive endoscopic papillectomies in FAP patients partially with large non-ampullary components for technical and clinical success and adverse event rates.

## Material and methods

### Patients and methods

A total of 40 papillectomies for tumors ≥ 10 mm measured by optical comparison to snares or biopsy forceps of the major papilla without prior endoscopic resections or papillotomies were performed in FAP patients at the Interdisciplinary Endoscopy Center at University Hospital Heidelberg between 2017 and 2024. Five patients were referred to our endoscopic unit for this procedure. Biopsies prior to planned resections were omitted to avoid fibrosis of the lesion. EUS was performed in selected patients with adenoma > 2 cm or suspicion of malignancy based on macroscopic evaluation. All procedures were performed under sedation with propofol, remifentanil, midazolam and/or ketamine. The retrospective data analysis was approved by Heidelberg Ethics Committee (S-871-2021). Written informed consent was obtained from each patient.

### Papillectomy

**Fig. 1:**
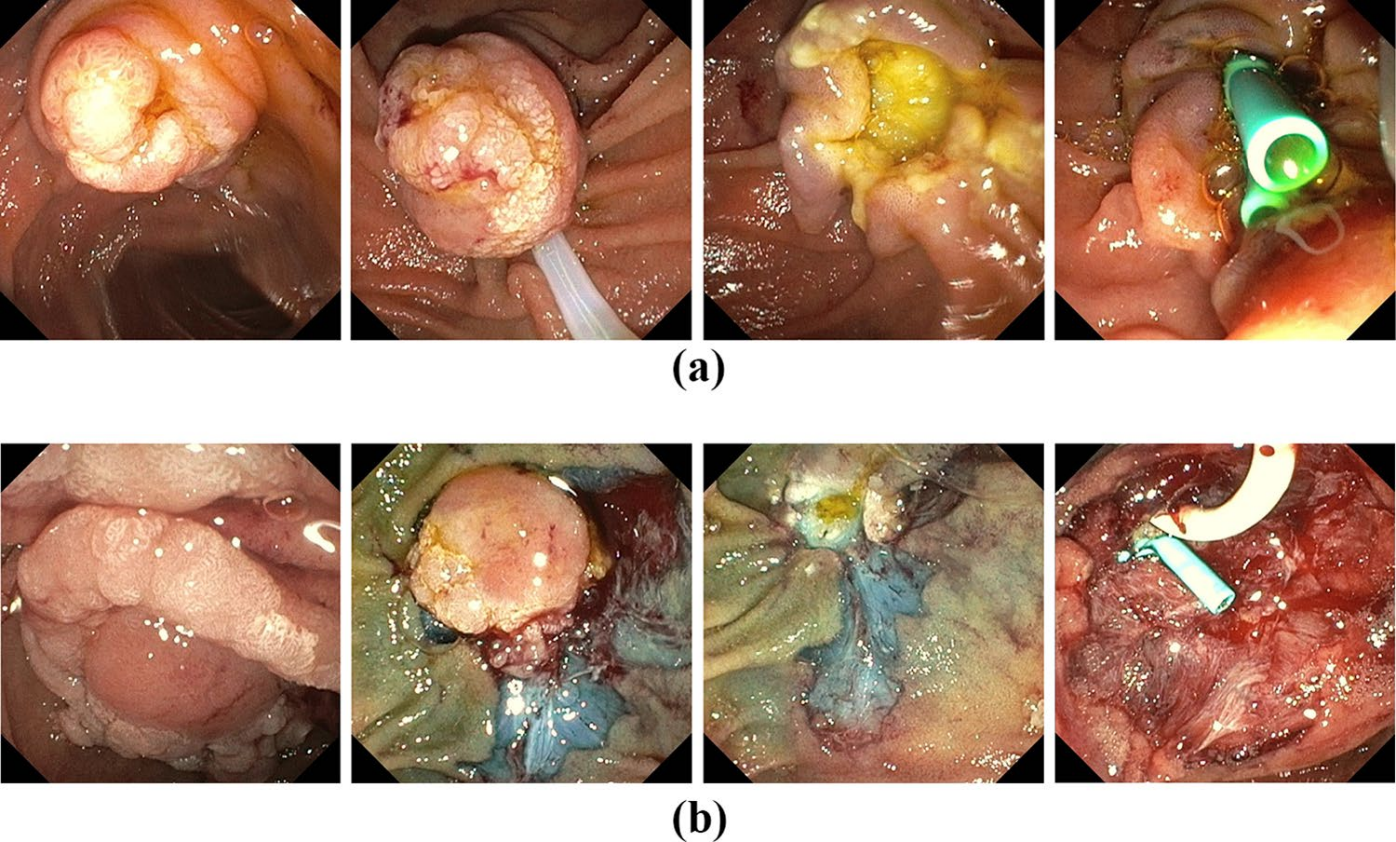
Examples of endoscopic papillectomies without (**A**) or with (**B**) laterally spreading adenoma. In all cases, temporary stenting of the bile and pancreatic duct was attempted after papillectomy to provide secured ducts in case of secondary endoscopic interventions for delayed bleeding and reduce the risk of pancreatitis. Stents were removed within 2 days after an uneventful resection. For laterally spreading adenoma, EMR and/or CSR of the lateral adenoma was performed prior to ampullectomy. EMR, endoscopic mucosa resection; CSR, cold snare resection

Papillectomy was performed in prone position with a standard duodenoscope (Olympus, TJF Q190V) under fluoroscopic guidance. Lesion size was estimated endoscopically in comparison to the size of the snare. Isolated ampullary adenomas were resected en-bloc with a 15 mm monofil snare (polypectomy snare SD-990/15, Olympus, Hamburg, Germany) with pure cutting current (Endocut I Effect 1, Erbe Vio 200D) (Fig. 1a). After papillectomy the pancreatic and bile duct were cannulated with a guidewire (0.025/0.035 Inch, VisiGlide, Olympus, Hamburg, Germany) via an ECRP catheter (Contour ERCP Cannula, Boston Scientific, Galway, Ireland) or papillotome (Ultratome XL 5.5F, Boston Scientific, Spencer, USA), respectively, or by the double guidewire technique according to [13]. Insertion of a straight 5-6F 5-7 cm endoprosthesis (EndoStay, Pflugbeil, Zorneding, Germany) was intended for the pancreatic duct and a 10F 10 cm (EndoStay, Pflugbeil, Zorneding, Germany) straight endoprosthesis or a 10F 14 cm double pigtail endoprosthesis (GastroSoft Biliary Endoprothesis, OptiMed, Ettlingen, Germany) for the bile duct without prior sphincterotomy. For adenomas extending beyond the papilla the non-ampullary component was removed by cold snare resection (CSR) (12 mm CrossSnare ZERO, Fujifilm medwork, Höchstadt, Germany) or endoscopic mucosal resection (EMR) (10 mm SnareMaster, Olympus, Hamburg, Germany) after submucosal injection with suprarenin 1:100.000 containing 40 µg/ml indigocarmine prior to papillectomy (Fig. 1b). For hemostasis or prophylactic closure, 16 mm clips were used (Lockado, Microtech, Hamburg, Germany). Diclofenac suppository (100 mg) was administered unless contraindicated. Immediate bleeding was defined as any bleeding necessitating endoscopic intervention and was treated by electrocautery with the tip of the snare, by APC (FiAPC 2200SC, Erbe, Tuebingen, Germany), local hemostyptic (PuraStat, 3-D Matrix Europe SAS, Caluire et Cuire, France) or clip application after pancreatic and bile duct stenting. Large resection surfaces were sealed with EndoClot (EndoClot Plus Co, Olympus, Hamburg, Germany) when high risk of delayed bleeding was assumed. Delayed bleeding was defined as endoscopically visible bleedings from the resection site after initial endoscopy had been completed. Pancreatitis was defined according to the revised Atlanta criteria fulfilling two from the following three criteria: typical abdominal pain, elevation of serum lipase of ≥ threefold of the upper limit of normal and evidence by imaging, respectively [14]. 100 mg diclofenac suppository was administered to all patients without contraindications e.g. impaired renal function or allergies.

### Follow-up

Unless delayed bleeding required endoscopic treatment, prophylactic plastic stents and pigtails were removed 2–3 days after papillectomy and sedation was offered to all patients. Initial follow-ups were scheduled 3 and 9 months after papillectomy followed by annual inspection with a duodenoscope or a cap-mounted gastroscope unless adenoma recurrence required repeated treatment by cold snare resection, EMR or APC treatment. Minimal tissue formation at the ampullary orifice was biopsied to rule out adenoma recurrence. Suspected intraductal recurrence was treated by radiofrequency ablation (Habib EndoHPB 8F, Boston Scientific, Malborough, USA) followed by prophylactic endoprosthesis placement for 3 months to prevent stricture formation of the bile duct.

### Statistical analysis

Categorical variables were presented as counts and proportions. Age was presented as mean and standard deviation. Univariate analysis was performed by chi-square test in case of binary variables, with t-test by age and body mass index (BMI). Binary multivariable logistic regression models were used to calculate adjusted analyses of bleeding complications and acute pancreatitis after papillectomy in the multivariate analysis. Variables with univariate *P*-values ≤ 0.2 were included in these analyses. Absolute numbers of events have been reported, and statistical significance was determined as *P* < 0.05. Statistical analyses were performed with the software SAS (Statistical Analysis System). Due to the nature of the study all *P*-values are reported as descriptive values and have no confirmatory value.

## Results

**Fig. 2:**
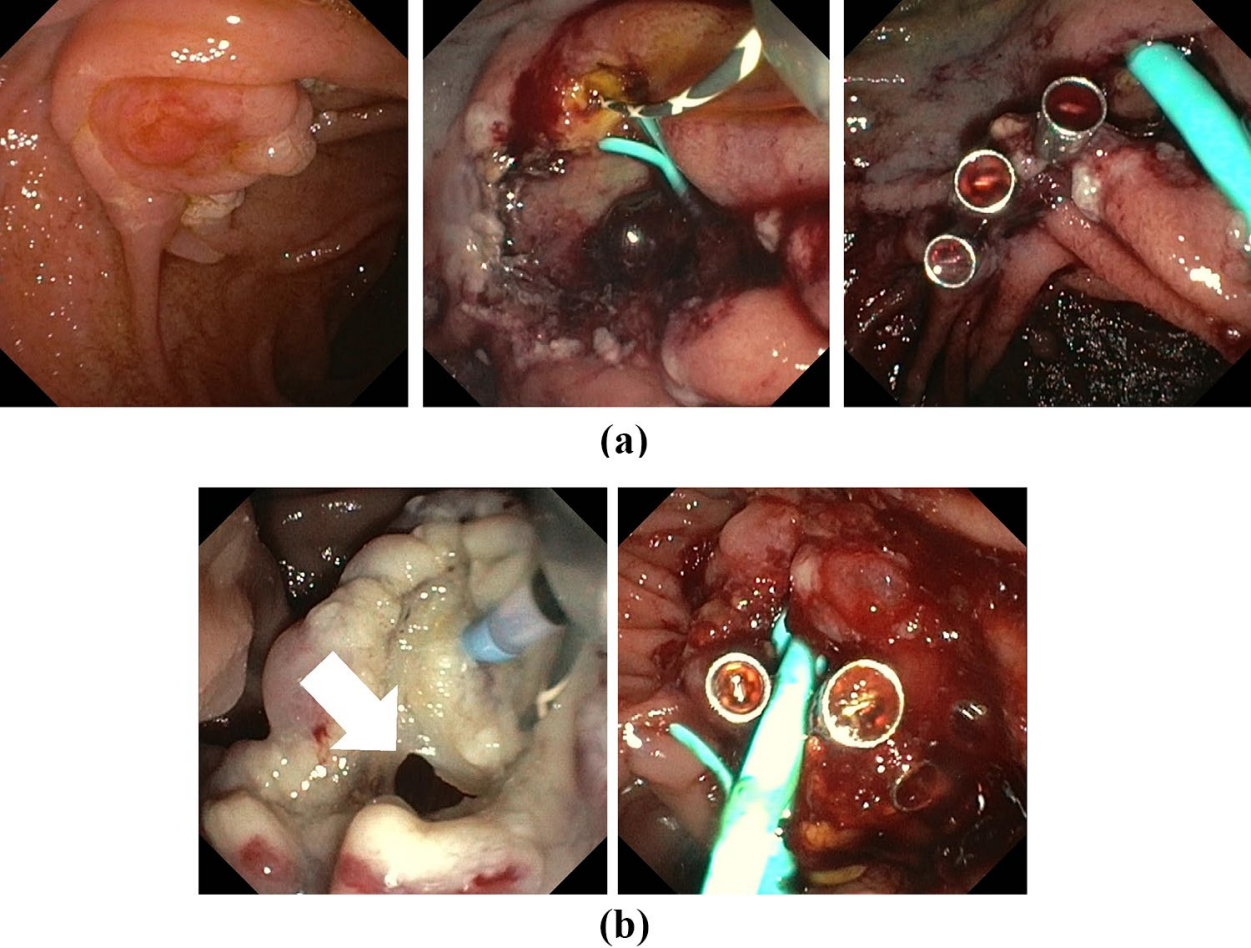
Examples of prophylactic clipping of large resection surface (**A**) and therapeutic clipping in one case of immediate perforation (**B**)

Papillectomy was performed in 21 male and 19 female patients with a mean age of 48 ± 14.7 years. Patient characteristics are presented in Table 1. Mean adenoma size was 14 mm with a range of 10-40 mm with an intraductal component in 5 cases (12.5%) measured by EUS < 10 mm. One example of the procedure is depicted in Fig. 1a. Laterally spreading adenoma occurred in n = 18 patients (45%), necessitating an additional resection by EMR/CSR (Fig. 1b). Prophylactic closure of large resection areas with clips was performed in n = 4 patients (Fig. 2a).

**Table 1:** Characteristics of patients, procedures and complications

*Characteristics*		
Sex	Male Female	n = 21 (53%) n = 19 (47%)
Age	Mean Standard deviation	48 years 14.7 years
Adenoma size (mm)	10 11–20 21–30 31–40	n = 18 (45%) n = 18 (45%) n = 3 (7.5%) n = 1 (2.5%)
*Procedure*		
Additional resection	Overall Cold snare Hot snare	n = 18 (45%) n = 14 (35%) n = 8 (20%)
Additional ablation	Overall Snare tip coagulation Argon plasma coagulation	n = 11 (28%) n = 11 (28%) n = 1 (3%)
Stent insertion	Pancreatic endoprosthesis Bile duct endoprosthesis	n = 31 (78%) n = 27 (68%)
Bleeding prophylaxis	Hemostyptic Clip	n = 5 (13%) n = 4 (10%)
*Complications*		
Immediate complications	Immediate Bleeding Perforation Sedation complication	n = 13 (33%) n = 1 (3%) n = 1 (3%)
Delayed complications	Delayed bleeding Delayed perforation (covered) Pancreatitis	n = 5 (13%) n = 1 (3%) n = 10 (25%)
Complication treatment	Clip application Hemostyptic Snare tip coagulation Blood transfusion	n = 7 (18%) n = 5 (13%) n = 3 (8%) n = 1 (3%)

Immediate bleeding occurred in 33% (n = 13) of which 54% (n = 7) needed clipping, 38% (n = 4) local hemostyptic, and 23% (n = 3) electrocautery. Immediate perforation was seen in 1 patient and was treated endoscopically (Fig. 2b). Delayed bleeding occurred in 13% (n = 5), which could be managed conservatively with only 1 patient requiring blood transfusion. In-house standard care of procedure included the attempt to insert a biliary endoprosthesis, which was successful in 68% of patients (n = 27), to enable safe application of clips in the event of acute bleeding without closing the main biliary duct. Protective pancreatic endoprosthesis could be placed in 78% (n = 31). Mild acute pancreatitis was observed in 25% (n = 10), was more frequent after additional EMR/CSR (28%) than without (18%) and could be managed conservatively in all cases.

Univariate regression analysis showed a significant higher immediate and late bleeding risk for men and after insertion of a protective pancreatic endoprosthesis (Table 2). Multivariate analysis showed non-significant trends for both parameters. The risk of acute pancreatitis was significantly higher in women with a non-significant trend in patients with additive CSR on univariate analysis. Multivariate analysis showed a significant higher risk for acute pancreatitis for women and after additional CSR (Table 3).

**Table 2:** Overall bleeding

Variables	Bleeding		*P*-value	
	Yes	No	Univariate	Multivariate
*Age (years)*				
	47 ± 14.7	48 ± 14.9	0.851	
*BMI (kg/m* ^*2*^ *)*				
	26.6 ± 6.8	27.5 ± 4.8	0.671	
*Sex*				
Female	4	15		
Male	12	9	**0.020**	0.056
*Adenoma size*				
10 mm	7	11		
> 10 mm	9	13	0.897	
*Additional EMR*				
No	14	18		
Yes	2	6	0.332	
*Additional CSR*				
No	12	14		
Yes	4	10	0.279	
*MBD endoprothesis*				
No	2	7		
Yes	14	17	0.216	
*MPD endoprothesis*				
No	2	11		
Yes	14	13	**0.027**	0.064
*Intraductal adenoma*				
No	14	21		
Yes	2	3	1.000	
*Coagulation*				
No	12	17		
Yes	4	7	0.773	

Univariate regression analysis showed a significant higher immediate and late bleeding risk for men and after insertion of a protective pancreatic endoprosthesis. Multivariate analysis showed non-significant trends for both parameters. EMR, endoscopic mucosa resection; CSR, cold snare resection

**Table 3:** Acute pancreatitis

Variables	Pancreatitis		*P*-value	
	Yes	No	Univariate	Multivariate
*Age (years)*				
	42 ± 9.0	49 ± 15.8	0.153	0.069
*BMI (kg/m* ^*2*^ *)*				
	27.3 ± 5.3	25.7 ± 8.1	0.476	
*Sex*				
Female	8	11		
Male	2	19	**0.018**	**0.031**
*Adenoma size*				
10 mm	6	12		
> 10 mm	4	18	0.271	
*Additional EMR*				
No	8	24		
Yes	2	6	1.000	
*Additional CSR*				
No	4	22		
Yes	6	8	0.055	**0.034**
*MBD endoprothesis*				
No	3	6		
Yes	7	24	0.512	
*MPD endoprothesis*				
No	4	9		
Yes	6	21	0.559	
*Intraductal adenoma*				
No	9	26		
Yes	1	4	0.783	
*Intraprocedural bleeding*				
No	9	18		
Yes	1	12	0.079	0.772
*Delayed bleeding*				
No	9	26		
Yes	1	4	0.783	
*Coagulation*				
No	6	23		
Yes	4	7	0.307	

Acute pancreatitis was significantly more common in women with a non-significant trend in patients with additive CSR on univariate analysis. Multivariate analysis showed a significant higher risk for acute pancreatitis for women and after additional CSR. EMR, endoscopic mucosa resection; CSR, cold snare resection

**Fig. 3:**
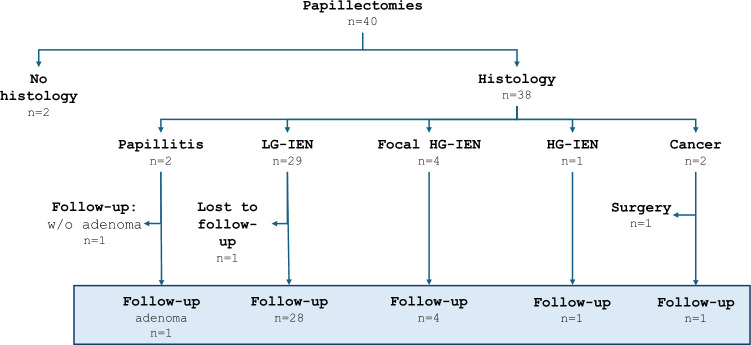
Flowchart of patients included in the follow-up for adenoma recurrence (grey box). Patients with no available histology, without adenoma, lost-to-follow-up patients and one operated patient were excluded from the analysis of adenoma recurrence. LG-IEN, low grade intraepithelial neoplasia; HG-IEN high grade intraepithelial neoplasia

Resected specimens could not be retrieved in n = 2 patients, which were excluded from the adenoma recurrence rate analysis. Histopathology of the remaining 38 specimens revealed papillitis in n = 2 patients, adenoma with low grade dysplasia in n = 29, focal high-grade dysplasia in n = 4, high-grade dysplasia in n = 1 and adenocarcinoma in n = 2 patients (Fig. 3). One of the 2 patients with papillitis showed adenoma recurrence on first endoscopic follow-up, suggesting a false-negative histology of the papillectomy. This patient was included in the follow-up analysis. The other patient with papillitis was excluded. One patient with low-grade dysplasia was lost to follow-up. One of the two cancer patients presented with a 10 mm lesion, where prior external biopsy reported a low-grade intraepithelial dysplasia and a 5 mm intra-ductal extension in EUS. This patient underwent a Whipple procedure for endoscopically incomplete tumor resection (final diagnosis after surgery: adenocarcinoma pT1b, pN0 (0/41), L0, V0, Pn0, R0) and was excluded from the follow-up analysis of local recurrence. A 34 months follow-up did not proof any cancer relapse so far. The other cancer patient (endoscopic resection of a 25 mm lesion without intra-ductal extension on EUS; histopathological report: pT1b, cN0, cM0, L0, V0, Pn0, R0) refused an operation and remained under endoscopic surveillance. CT-scan 22 months after the papillectomy did not show any signs of metastatic disease. Finally, n = 35 patients could be included in the follow-up analysis of recurrence (Fig. 3). Of these, recurrence-free resection could be achieved after one single papillectomy session in 74% (n = 26/35), with a median follow-up of 368 days (range 15–832), although 46% (n = 11/24) of these papillectomies were combined with CSR and/or EMR. Only 31% (n = 11/35) of patients developed a histologically confirmed adenoma recurrence after a median time interval of 89 days (range 30–1204), although, the two patients with the longest time interval to relapse (463 days, 1204 days) neglected close-meshed surveillance for 1 and 3 years, respectively. Adenoma recurrences could be successfully treated by EMR, CSR, argon plasma coagulation and/or radio frequency ablation in 36% (n = 4/11) by 1 and in another 46% (n = 5/11) by 2 additional procedures. Thus, cumulative recurrence-free resection was achieved in 94% (n = 33/35) after a mean of 1.4 procedures at a median follow-up of 591 days (range 15–2605). None of the analyzed variables were associated with the risk of adenoma recurrence on uni- or multivariate analysis.

## Discussion

FAP patients undergo a plethora of surgical and endoscopic procedures during their lifetime. Still, these patients are under perpetual risk of cancer formation, especially from duodenal and gastric adenomas. While some guidelines consider each ampullary adenoma as a candidate for endoscopic resection especially when exceeding 10 mm or with adverse histology [6], others recommend endoscopic resection only for a size ≥ 10 mm and with excessive growth [5]. These conflicting recommendations reflect the difficult balance between the avoidance of malignant progression versus periprocedural complications, mainly bleeding, perforation and acute pancreatitis. However, symptomatic ampullary adenomas with recurrent pancreatitis might justify even early endoscopic papillectomy [15], although recurrent pancreatitis might occur in FAP patients even without pancreatic obstruction by ampullary adenoma [16]. For an informed choice regarding papillectomy in FAP patients more data are needed about the success and complication rate and about risk factors for complications.

In our series, the percentage of laterally spreading adenomas was 45%, necessitating extensive additional resections by EMR and/or CSR for adenomas of up to 40 mm of size. Although a two-stage endoscopic resection has been recently published for large laterally spreading ampullary adenoma [10], we performed all combined resections in one single session. In these cases, we prefer to resect the lateral adenoma first, leaving the exposed papilla for final snare resection (Fig. 1b), although this might harbor a higher risk for perforation. However, our immediate perforation risk (2.5%) was comparable to pooled data from mixed cohorts (3.1%) [17] and FAP subgroups (2–3%) [3, 4], while a recent study reported a perforation rate of 21% [18].

Initial R0 resection rate of endoscopic papillectomy seem to be lower in FAP patients than in sporadic ampullary adenoma (64% versus 83%) [4] and as low as 36% when same-session adjunctive therapies were needed for complete resection [17]. Thus, high technical success rates of 95% in FAP patients in small case series might reflect a favorable patient selection [7], as larger series reported recurrence rates of only 48% in FAP patients [3]. Accordingly, in our series, 69% recurrence-free resection was achieved after a single session and 94% after repeated endoscopic resection and ablation techniques. This is in accordance with other studies reaching final recurrence-free resection after repeated interventions in 93% of FAP patients [4]. Many parameters have been discussed as risk factors for recurrence: genetic background (FAP), bile duct dilatation, periampullary resection, piece meal resection, adenoma size, and intraductal invasion [3, 7, 17]. Limited by our case number, none of these parameters were significantly associated with adenoma recurrence.

Intraprocedural bleeding was the most frequent immediate adverse event (33%) in our series and significantly associated with male sex. The association with insertion of a protective pancreatic endoprosthesis seems to represent a meta effect. Reported bleeding rates were much less and ranged between 11 and 13% [3, 4, 17]. In these studies bleeding was either defined by the established lexicon by the ASGE [19], which neglects transient bleeding episodes, or were not specified. Thus, our definition of bleeding with each event requiring an endoscopic hemostasis, has a lower threshold. In contrast, Le Bras et al. report a delayed bleeding episode after papillectomy in 26% of their patients [18] reflecting a twofold higher rate compared to our cohort.

Acute pancreatitis has been reported in 11–17% of papillectomies in FAP patients [3, 4] but cited with up to 20% in endoscopic guidelines [5]. We found an overall rate of pancreatitis of 25%, which was higher in patients with additional EMR/CSR (28%) than in single papillectomies (18%). However, in multivariate regression analysis, female sex and CSR were significantly associated with acute pancreatitis. This finding is in accordance with a 2015 published meta-analysis of 28 studies demonstrating an odds ratio of 1.46 for females regarding the post-ERCP pancreatitis rate [20, 21]. Whether female patients might benefit from a more aggressive fluid substitution as a prophylaxis for acute pancreatitis, needs to be investigated in prospective trials. BMI was not associated with the risk of acute pancreatitis. Although their protective effect is well established in larger cohorts [17], in our small series main pancreatic duct stenting was not associated with a lower risk of pancreatitis. However, prophylactic pancreatic stenting was technically successful in only 78%.

Long-term recurrence-free resection after a single procedure was high (74%) and nearly all relapsed adenoma could be endoscopically resected with a maximum of 2 additional interventions. Only 2 patients (6%) were not free of adenoma at the end of the surveillance. However, in one of these patients follow-up after therapeutic biopsy of a small adenoma relapse is still pending. Our recurrence rates are within the range of reported results of 21–48% [3, 4]. Despite repeated adenoma resection, recurrence forced surgery as a definite therapy in 2–13% of reported cohorts [3, 22]. In our cohort, none of the patients needed rescue surgery for adenoma recurrence so far. Known risk factors for adenoma recurrence are intraductal adenoma growth, bile duct dilation, and periampullary adenoma spread [3]. Due to the limited number of our cases, we could not confirm these risk factors on uni- or multivariate regression analysis.

Ampullary carcinoma are rarely found in the cohort of adenomas considered suitable for endoscopic resection (0–2%) but constituted 5% of our cohort. While one of these patients underwent curative rescue surgery, the other patient denied surgery and is still asymptomatic 746 days after endoscopic resection. Interestingly, while one carcinoma was detected within a 25 mm adenoma, the other one was found in a small ampullary lesion of 10 mm.

Endoscopic papillectomy shows high clinical success in FAP patients even with laterally spreading adenoma. Adenoma recurrence can be successfully treated with a limited number of reinterventions. Severe adverse events are rare and endoscopically manageable. In our cohort the most common additional resection technique was cold snare resection which might explain the higher immediate bleeding rate compared to other reports. However, the perforation and delayed bleeding rates reflecting complications with a more relevant clinical impact were much lower than recently published. Since female patients showed a higher risk for acute pancreatitis, these patients might benefit even more from prophylactic endoprosthesis application, non-steroidal antiphlogistics or forced intravenous hydration. As one carcinoma was detected in a 10 mm specimen with an intra-ductal extension of 5 mm, current thresholds for endoscopic papillectomy might be too strict for selected patients.

## Data Availability

Data will be made available on request by the corresponding author.
